# Supplementary material for: Temporal microstructure of dyadic social behavior during relationship formation in mice
Source: PLoS One. 2019 Dec 10;14(12):e0220596. doi: 10.1371/journal.pone.0220596 (PMC6903754; doi:10.1371/journal.pone.0220596)
Supplement: S1 Fig — (PDF) [file pone.0220596.s001.pdf]

S1 Fig. The durations of investigative and social behaviors exhibited by each mouse in each dyad across 5 days of observation.

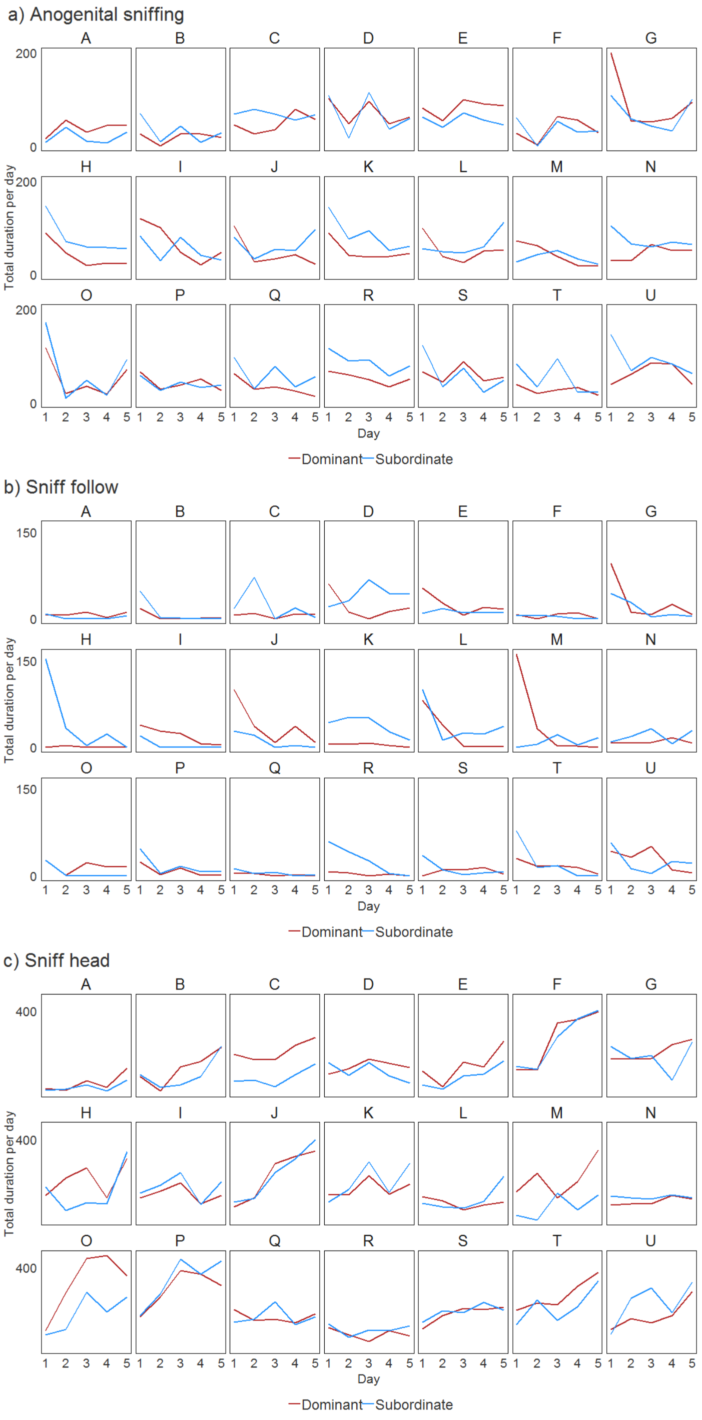

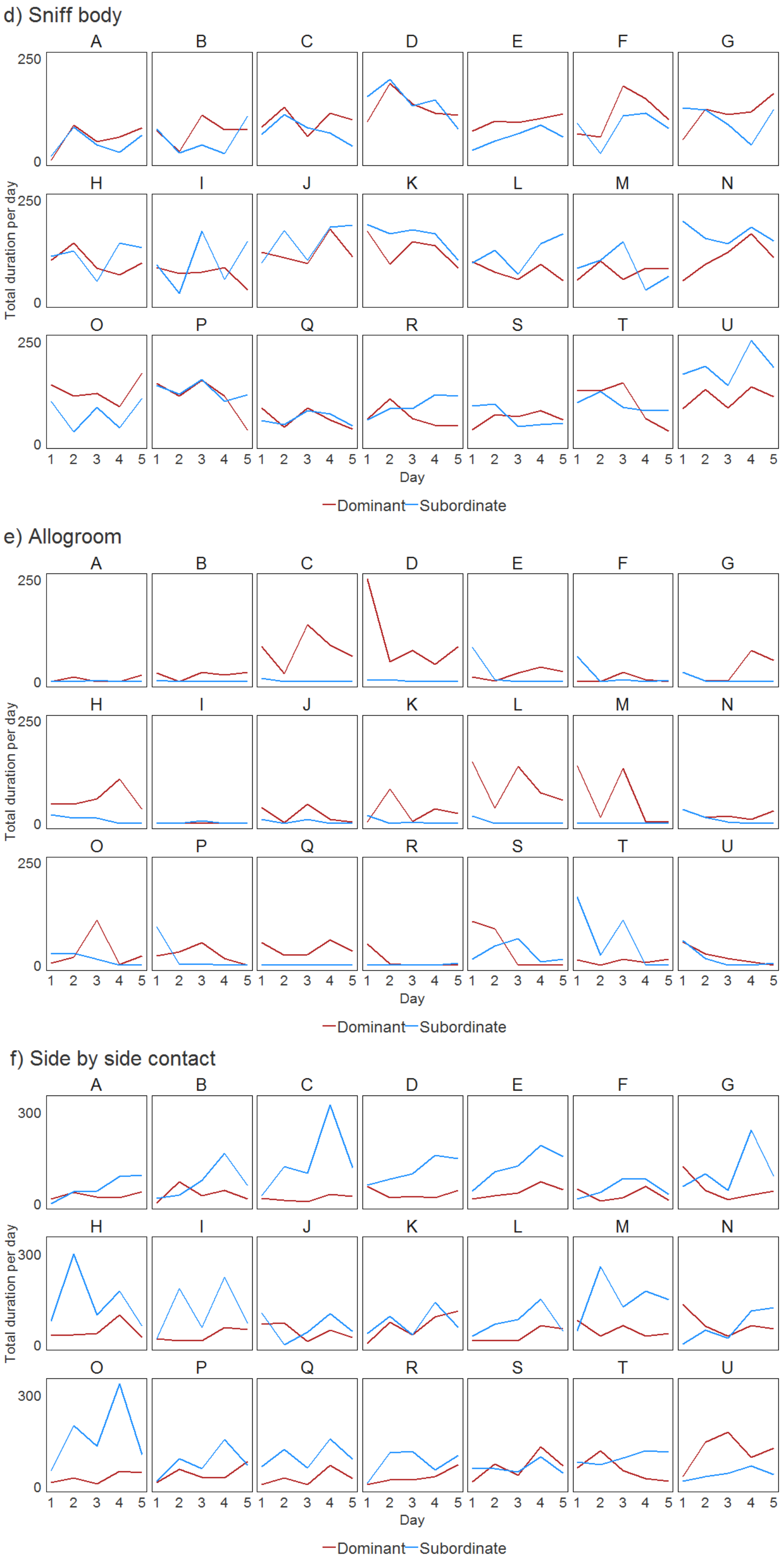
d) Sniff body
A B C D E F G H I J K L M N O P Q R S T U
Total duration per day
250
0
Day
1 2 3 4 5
Dominant Subordinate
e) Allogroom
A B C D E F G H I J K L M N O P Q R S T U
Total duration per day
250
0
Day
1 2 3 4 5
Dominant Subordinate
f) Side by side contact
A B C D E F G H I J K L M N O P Q R S T U
Total duration per day
300
0
Day
1 2 3 4 5
Dominant Subordinate
