# Supplementary material for: Temporal microstructure of dyadic social behavior during relationship formation in mice
Source: PLoS One. 2019 Dec 10;14(12):e0220596. doi: 10.1371/journal.pone.0220596 (PMC6903754; doi:10.1371/journal.pone.0220596)
Supplement: S2 Fig — Temporal pattern of aggressive (blue) and subordinate (red) behaviors by the eventual dominant (D) and subordinate (S) males on days 1–5 of all 21 dyads. Yellow bars indicate aggressive/subordinate bursts identified by the Kleinburg Burst Detection algorithm (gamma = 0.3). (PDF) [file pone.0220596.s002.pdf]

S2 Fig. Temporal pattern of aggressive (blue) and subordinate (red) behaviors by the eventual dominant (D) and subordinate (S) males on days 1-5 of all 21 dyads. Yellow bars indicate aggressive/subordinate bursts identified by the Kleinburg Burst Detection algorithm (gamma=0.3).

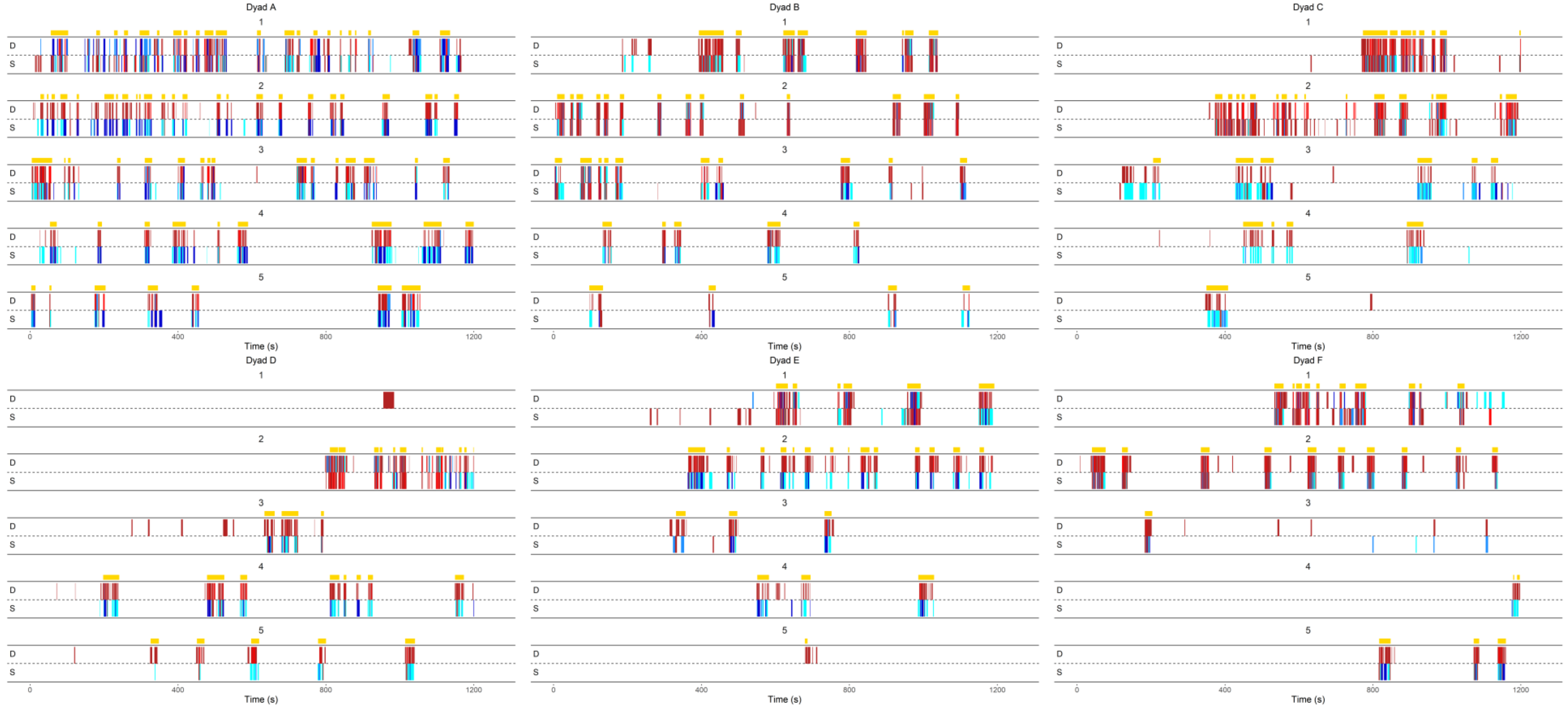

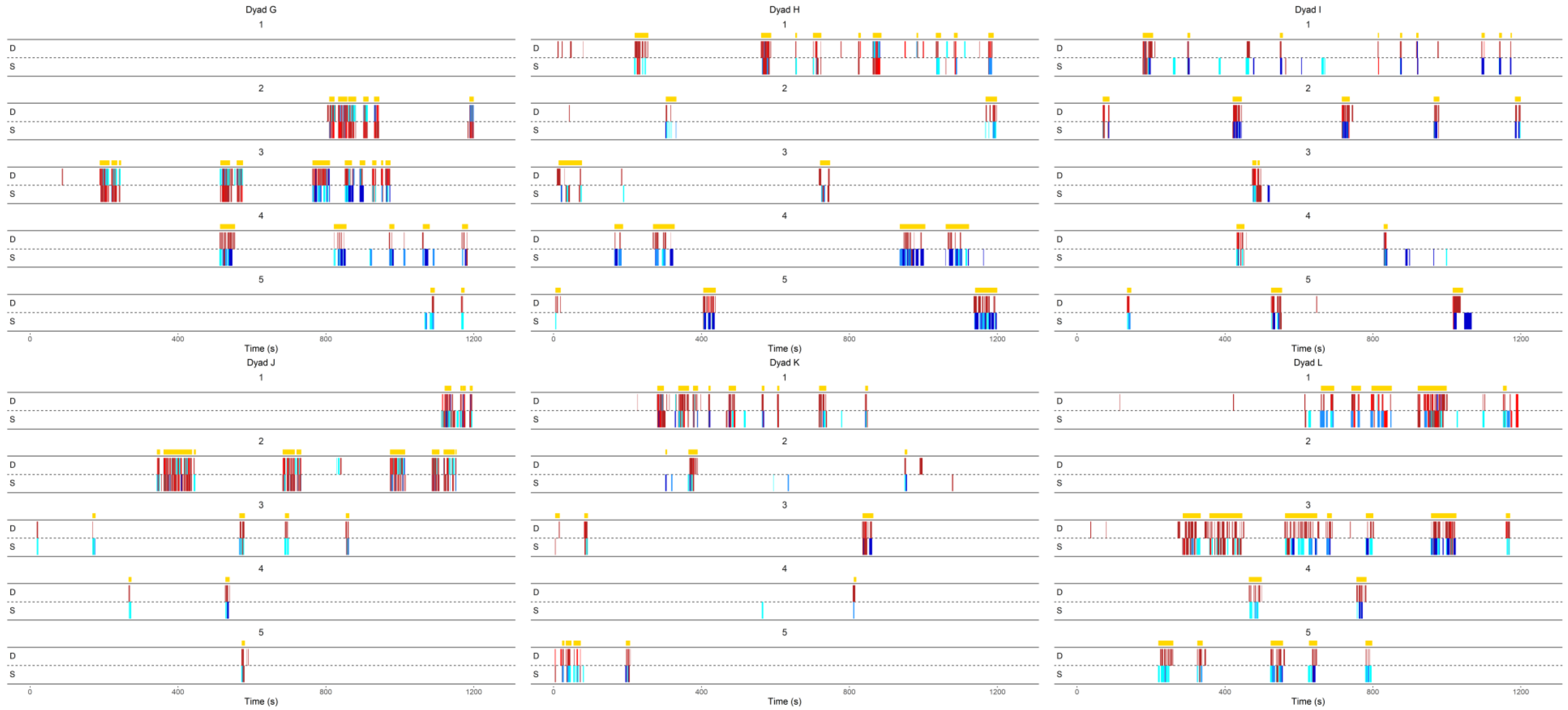
Dyad G
Dyad H
Dyad I
Dyad J
Dyad K
Dyad L
1
2
3
4
5
D
S
0
400
800
1200
Time (s)

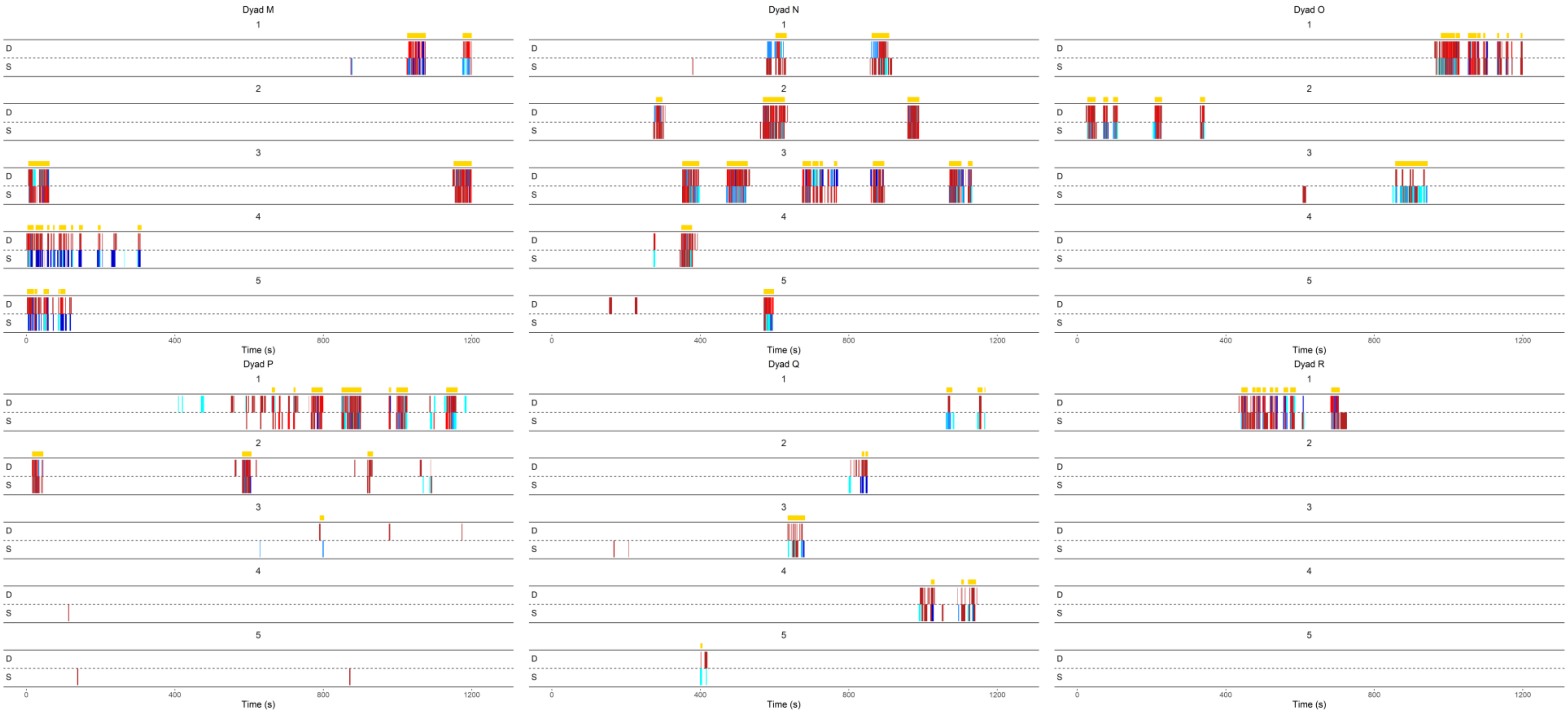
Dyad M
Dyad N
Dyad O
Dyad P
Dyad Q
Dyad R
1
2
3
4
5
D
S
0
400
800
1200
Time (s)

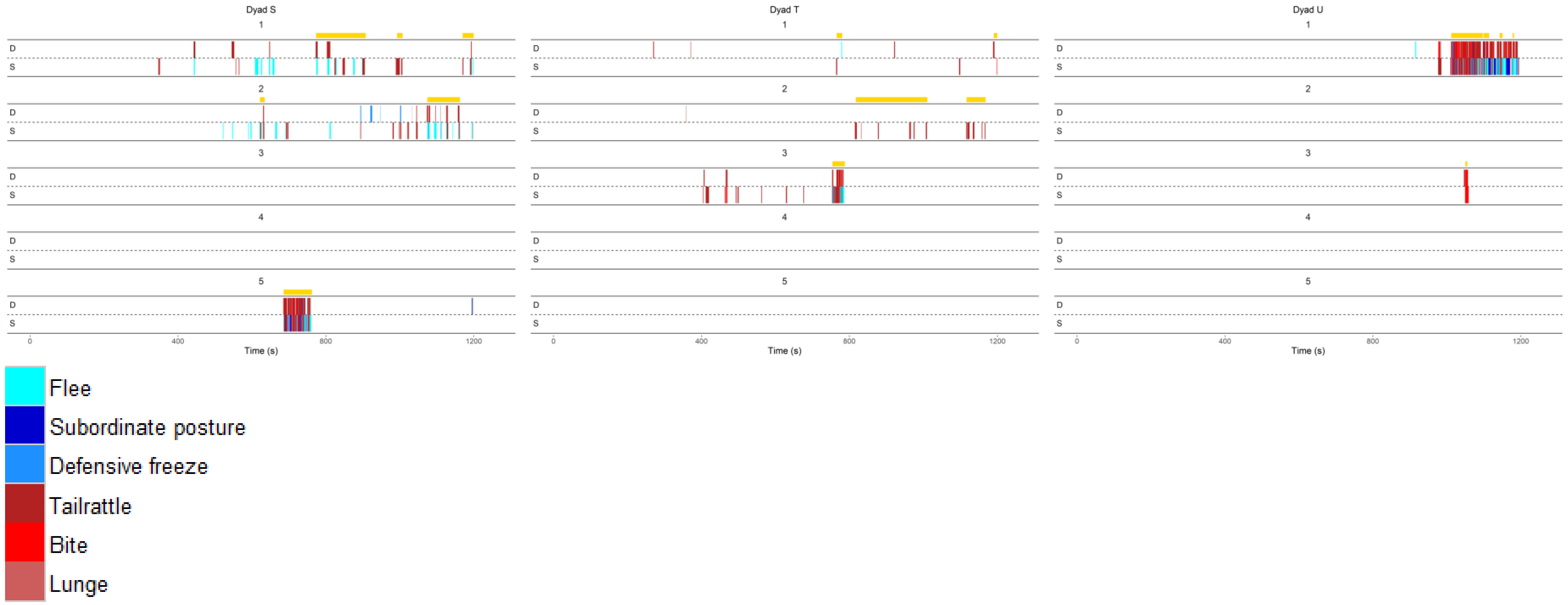

Dyad S
Dyad T
Dyad U
1
2
3
4
5
D
S
0
400
800
1200
Time (s)
Flee
Subordinate posture
Defensive freeze
Tailrattle
Bite
Lunge
