# Supplementary material for: Temporal microstructure of dyadic social behavior during relationship formation in mice
Source: PLoS One. 2019 Dec 10;14(12):e0220596. doi: 10.1371/journal.pone.0220596 (PMC6903754; doi:10.1371/journal.pone.0220596)
Supplement: S1 Table — The value in parenthesis is p-value and asterisks indicate; *: p<0.05, **: p<0.01, ***: p<0.001. (PDF) [file pone.0220596.s003.pdf]

**S1 Table. The difference in durations of behaviors between dominant and subordinate mice across three phases (paired Wilcoxon Signed Rank Sum Tests).** The value in parenthesis is p-value and asterisks indicate; *: p<0.05, **: p<0.01, ***: p<0.001

| State | Behavior | Pre-resolution | Middle | Post-resolution |
|---|---|---|---|---|
| Aggressive Behaviors | lunge | V= 60.5 (p= 0.638) | 123 (0.005)** | 206 (0.000)*** |
| | bite | 69 (0.629) | 108 (0.041)* | 186 (0.000)*** |
| | tail rattle | 34 (0.148) | 97 (0.344) | 202 (0.000)*** |
| Subordinate Behaviors | flee | 70.5 (0.569) | 20 (0.007)** | 0 (0.000)*** |
| | defensive freeze | 61 (0.616) | 22 (0.011)* | 3 (0.000)*** |
| | subordinate posture | 52 (1.000) | 15 (0.020)* | 0 (0.000)*** |
| Investigative Behaviors | sniff head | 155 (0.175) | 58 (0.623) | 145 (0.140) |
| | sniff body | 106 (0.754) | 55 (0.320) | 117 (0.668) |
| | sniff anogenital | 72 (0.135) | 33 (0.074) | 64 (0.131) |
| | sniff follow (sniffing while following) | 84 (0.281) | 40 (0.154) | 101 (0.896) |
| Other Social Behaviors | pursue (without sniffing) | 56 (0.842) | 121 (0.005)** | 210 (0.000)*** |
| | allogroom | 111 (0.275) | 78 (0.002)** | 198 (0.001)** |
| | side by side contact | 108 (0.808) | 34 (0.047)* | 11 (0.000)*** |
| Repetitive Behaviors | digging | 122 (0.033)* | 103 (0.015)* | 205 (0.000)*** |
| | self-grooming | 112 (0.507) | 44 (0.944) | 112 (0.808) |
| Activity | moving | 129 (0.651) | 119 (0.047)* | 138 (0.225) |
| | rearing | 125 (0.754) | 91 (0.508) | 126 (0.444) |
| | jumping | 4 (0.773) | 3 (0.233) | 86 (0.109) |
| Inactivity | idle/nothing | 79.5 (0.350) | 51 (0.235) | 14 (0.001)** |
