# Supplementary material for: Temporal microstructure of dyadic social behavior during relationship formation in mice
Source: PLoS One. 2019 Dec 10;14(12):e0220596. doi: 10.1371/journal.pone.0220596 (PMC6903754; doi:10.1371/journal.pone.0220596)
Supplement: S2 Table — Asterisks indicate; **: p<0.01, ***: p<0.001. We conservatively considered the transitions to be significant if p≤0.01 and only significant p-values of transitions are displayed. (PDF) [file pone.0220596.s004.pdf]

S2 Table. The median probability of behavioral transition within individuals and its significance tested based on permutation.
Asterisks indicate; **: p<0.01, ***: p<0.001. We conservatively considered the transitions to be significant if p≤0.01 and only significant p-values of transitions are displayed.

Contingency	Pre-resolution		Post-resolution	
	Dominants	Subordinates	Dominants	Subordinates
moving → rearing	0.422***	0.407 ***	0.412 ***	0.455 ***
moving → digging	0.084 ***	0.053 **	0.108 ***	0.033 **
sniff head → sniff body	0.385 ***	0.417 ***	0.276 ***	0.281 ***
tailrattle → bite	0.258 **	0.250 ***	0.264 ***	0.078 **
bite → tailrattle	0.455 ***	0.565 ***	0.762 ***	0.345 ***
rearing → moving	0.800 ***	0.747 ***	0.704 ***	0.671 ***
sniff body → sniff anogenital	0.250 ***	0.298 ***	0.215 ***	0.209 ***
sniff anogenital → sniff body	0.300 ***	0.250 **	0.256 ***	0.221 **
tailrattle → lunge	0.046 ***	0.063 ***	0.116 ***	
sniff body → sniff head	0.217 **	0.162 **	0.318 ***	
sniff anogenital → sniff follow (sniffing while following)	0.100 ***	0.098 **	0.051 ***	
lunge → tailrattle	0.274 ***	0.238 ***	0.511 ***	
lunge → bite	0.167 **		0.320 ***	
tailrattle → defensive freeze	0.094 **			0.184 ***
defensive freeze → tailrattle	0.400 **			0.358 ***
sniff follow (sniffing while following) → sniff body	0.250 **			
moving → self-grooming			0.024 ***	
sniff head → rearing			0.149 ***	
sniff head → allogroom			0.020 **	
tailrattle → pursue (without sniffing)			0.176 ***	
tailrattle → allogroom			0.107 ***	
rearing → sniff head			0.168 ***	
rearing → contact side by side (without sniffing)			0.053 ***	

Contingency	Pre-resolution		Post-resolution	
	Dominants	Subordinates	Dominants	Subordinates
contact side by side (without sniffing) → rearing			0.149 ***	
sniff body → moving			0.283 ***	
sniff body → rearing			0.093 ***	
sniff anogenital → rearing			0.101 ***	
self-grooming → rearing			0.223 ***	
digging → moving			0.280 ***	
digging → sniff head			0.210 ***	
digging → rearing			0.301 ***	
pursue (without sniffing) → tailrattle			0.613 ***	
allogroom → tailrattle			0.345 ***	
sniff head → contact side by side (without sniffing)				0.139 **
tailrattle → flee				0.318 ***
tailrattle → subordinate posture				0.143 **
flee → tailrattle				0.277 ***
flee → defensive freeze				0.254 ***
flee → subordinate posture				0.093 **
defensive freeze → flee				0.333 ***
subordinate posture → tailrattle				0.257 **
subordinate posture → flee				0.287 ***
contact side by side (without sniffing) → idle/nothing				0.122 ***
