# Supplementary material for: Temporal microstructure of dyadic social behavior during relationship formation in mice
Source: PLoS One. 2019 Dec 10;14(12):e0220596. doi: 10.1371/journal.pone.0220596 (PMC6903754; doi:10.1371/journal.pone.0220596)
Supplement: S3 Table — The asterisks indicate p-values from paired Wilcoxon Signed Rank Tests; *: p<0.05, **: p<0.01, ***: p<0.001. The values in the parenthesis indicate V value from the tests. (PDF) [file pone.0220596.s005.pdf]

S3 Table. FSTTC values of each state transitions and the differences in FSTTC values between directions. The asterisks indicate p-values from paired Wilcoxon Signed Rank Tests; *: p<0.05, **: p<0.01, ***: p<0.001. The values in the parenthesis indicate V value from the tests.

**Contingency**	**Pre-resolution phase**		**Post-resolution phase**		**Wilcoxon Signed Rank Tests**	
	**DOM→SUB**	**SUB→DOM**	**DOM→SUB**	**SUB→DOM**	**Pre**	**Post**
lunge/bite → lunge/bite	0.45 ± 0.05	0.64 ± 0.06	0.21 ± 0.04	0.58 ± 0.04	-	DS<SD**(12)
lunge/bite → subordinate	0.41 ± 0.04	0.48 ± 0.04	0.75 ± 0.03	0.19 ± 0.05	-	DS>SD**(66)
lunge/bite → tailrattle	0.63 ± 0.04	0.62 ± 0.05	0.52 ± 0.07	0.68 ± 0.06	-	-
lunge/bite → pursuing	-0.01 ± 0.01	-0.02 ± 0.00	0.00 ± 0.00	0.14 ± 0.04	-	-
lunge/bite → allogroom	-0.04 ± 0.00	-0.02 ± 0.01	-0.01 ± 0.00	0.01 ± 0.01	-	-
lunge/bite → side by side	-0.05 ± 0.00	-0.04 ± 0.01	0.06 ± 0.05	-0.02 ± 0.00	DS<SD*(5)	-
lunge/bite → digging	-0.04 ± 0.00	-0.04 ± 0.00	-0.02 ± 0.00	-0.02 ± 0.00	-	-
lunge/bite → sniffing	-0.13 ± 0.01	-0.12 ± 0.01	-0.10 ± 0.01	-0.12 ± 0.00	-	-
subordinate → lunge/bite	0.48 ± 0.05	0.5 ± 0.05	0.49 ± 0.09	0.40 ± 0.05	-	-
subordinate → subordinate	0.25 ± 0.04	0.25 ± 0.06	0.56 ± 0.08	0.04 ± 0.01	-	DS>SD**(75)
subordinate → tailrattle	0.56 ± 0.05	0.59 ± 0.06	0.53 ± 0.09	0.58 ± 0.04	-	-
subordinate → pursuing	0.06 ± 0.02	0.12 ± 0.02	-	0.28 ± 0.04	-	-
subordinate → allogroom	-0.01 ± 0.01	-0.02 ± 0.00	0.02 ± 0.01	0.09 ± 0.02	-	-
subordinate → side by side	-0.03 ± 0.00	-0.02 ± 0.02	-0.02 ± 0.01	-0.03 ± 0.00	-	-
subordinate → digging	-0.03 ± 0.00	-0.04 ± 0.00	-0.01 ± 0.00	-0.04 ± 0.00	-	DS>SD**(77)
subordinate → sniffing	-0.12 ± 0.01	-0.09 ± 0.02	-0.07 ± 0.01	-0.10 ± 0.01	-	DS>SD*(66)
tailrattle → lunge/bite	0.42 ± 0.04	0.46 ± 0.04	0.12 ± 0.02	0.46 ± 0.05	-	DS<SD***(0)
tailrattle → subordinate	0.32 ± 0.04	0.32 ± 0.05	0.58 ± 0.03	0.10 ± 0.03	-	DS>SD**(77)
tailrattle → tailrattle	0.56 ± 0.04	0.52 ± 0.04	0.34 ± 0.05	0.60 ± 0.04	-	DS<SD**(20)
tailrattle → pursuing	-0.01 ± 0.01	0 ± 0.02	0.06 ± 0.02	0.19 ± 0.03	-	-
tailrattle → allogroom	-0.05 ± 0.00	-0.04 ± 0.01	-0.02 ± 0.00	0.05 ± 0.02	-	DS<SD**(2)
tailrattle → side by side	-0.04 ± 0.01	-0.05 ± 0.00	0.01 ± 0.01	-0.02 ± 0.00	-	-
tailrattle → digging	-0.05 ± 0.00	-0.05 ± 0.00	-0.03 ± 0.00	-0.03 ± 0.00	-	-
tailrattle → sniffing	-0.11 ± 0.01	-0.11 ± 0.01	-0.10 ± 0.01	-0.09 ± 0.01	-	-

Contingency	Pre-resolution phase		Post-resolution phase		Wilcoxon Signed Rank Tests	
	DOM→SUB	SUB→DOM	DOM→SUB	SUB→DOM	Pre	Post
pursuing → lunge/bite	0.34 ± 0.06	0.41 ± 0.08	0.12 ± 0.03	0.50 ± 0.15	-	-
pursuing → subordinate	0.40 ± 0.05	0.34 ± 0.09	0.72 ± 0.04	-	-	-
pursuing → tailrattle	0.48 ± 0.05	0.44 ± 0.09	0.33 ± 0.05	0.50 ± 0.16	-	-
pursuing → pursuing	-0.01 ± 0.00	-0.01 ± 0.00	0.00 ± 0.00	0.00 ± 0.00	-	-
pursuing → allogroom	-0.01 ± 0.00	0.00 ± 0.01	-0.01 ± 0.00	0.00 ± 0.00	-	-
pursuing → side by side	-0.01 ± 0.01	-0.01 ± 0.00	0.07 ± 0.03	0.49 ± 0.16	-	-
pursuing → digging	-0.01 ± 0.00	-0.01 ± 0.00	-0.01 ± 0.00	-0.02 ± 0.00	-	-
pursuing → sniffing	-0.1 ± 0.01	-0.07 ± 0.02	-0.1 ± 0.01	-0.11 ± 0.00	-	-
allogroom → lunge/bite	0.00 ± 0.01	-0.03 ± 0.00	0.00 ± 0.01	0.05 ± 0.04	-	-
allogroom → subordinate	0.06 ± 0.05	0.06 ± 0.04	0.27 ± 0.06	0.10 ± 0.04	-	-
allogroom → tailrattle	0.03 ± 0.03	0.07 ± 0.04	0.09 ± 0.03	0.05 ± 0.04	-	-
allogroom → pursuing	-0.01 ± 0.00	-0.01 ± 0.00	0.00 ± 0.00	-0.01 ± 0.00	-	-
allogroom → allogroom	-0.01 ± 0.00	-0.01 ± 0.00	0.00 ± 0.00	0.02 ± 0.02	-	-
allogroom → side by side	-0.01 ± 0.01	0.00 ± 0.01	0.09 ± 0.02	0.10 ± 0.05	-	-
allogroom → digging	-0.02 ± 0.00	0.07 ± 0.07	-0.01 ± 0.00	-0.02 ± 0.00	-	-
allogroom → sniffing	0.02 ± 0.06	-0.01 ± 0.03	-0.07 ± 0.02	-0.07 ± 0.02	-	-
side by side → lunge/bite	0.00 ± 0.02	-0.03 ± 0.01	-0.02 ± 0.00	-0.01 ± 0.01	DS>SD**(65)	-
side by side → subordinate	-0.02 ± 0.01	-0.03 ± 0.00	-0.02 ± 0.00	-0.04 ± 0.00	-	DS>SD*(68)
side by side → tailrattle	0.00 ± 0.03	-0.02 ± 0.01	-0.02 ± 0.00	0.01 ± 0.01	-	DS<SD**(22)
side by side → pursuing	-0.01 ± 0.00	-0.01 ± 0.00	-0.03 ± 0.00	-0.02 ± 0.00	-	-
side by side → allogroom	0.03 ± 0.03	0.02 ± 0.02	-0.01 ± 0.00	0.01 ± 0.01	-	-
side by side → side by side	0.04 ± 0.02	0.01 ± 0.01	0.07 ± 0.01	0.02 ± 0.01	-	DS>SD**(179)
side by side → digging	0.00 ± 0.01	-0.02 ± 0.01	-0.02 ± 0.00	-0.02 ± 0.00	-	-
side by side → sniffing	0.18 ± 0.07	0.28 ± 0.06	0.15 ± 0.02	0.16 ± 0.01	-	-
digging → lunge/bite	-0.04 ± 0.00	-0.04 ± 0.00	-0.02 ± 0.00	-0.02 ± 0.00	-	-
digging → subordinate	-0.04 ± 0.00	-0.03 ± 0.00	-0.04 ± 0.00	-0.01 ± 0.00	-	DS<SD**(1)

Contingency	Pre-resolution phase		Post-resolution phase		Wilcoxon Signed Rank Tests	
	DOM→SUB	SUB→DOM	DOM→SUB	SUB→DOM	Pre	Post
digging → tailrattle	-0.05 ± 0.00	-0.05 ± 0.00	-0.03 ± 0.00	-0.03 ± 0.00	-	-
digging → pursuing	-0.01 ± 0.00	-0.01 ± 0.00	-0.02 ± 0.00	-0.01 ± 0.00	-	-
digging → allogroom	-0.02 ± 0.00	-0.02 ± 0.00	-0.02 ± 0.00	-0.01 ± 0.00	-	-
digging → side by side	-0.02 ± 0.00	-0.02 ± 0.00	-0.03 ± 0.01	-0.02 ± 0.00	-	-
digging → digging	0.03 ± 0.01	0.05 ± 0.01	-0.01 ± 0.01	0.06 ± 0.04	-	-
digging → sniffing	0.10 ± 0.02	0.00 ± 0.02	0.06 ± 0.02	0.07 ± 0.03	DS>SD**(95)	-
sniffing → lunge/bite	-0.11 ± 0.01	-0.12 ± 0.01	-0.12 ± 0.00	-0.10 ± 0.01	-	DS<SD*(34)
sniffing → subordinate	-0.09 ± 0.02	-0.12 ± 0.02	-0.11 ± 0.01	-0.09 ± 0.01	-	-
sniffing → tailrattle	-0.09 ± 0.03	-0.12 ± 0.01	-0.11 ± 0.00	-0.11 ± 0.01	-	-
sniffing → pursuing	-0.11 ± 0.01	-0.11 ± 0.01	-0.11 ± 0.00	-0.10 ± 0.01	-	-
sniffing → allogroom	-0.10 ± 0.01	-0.11 ± 0.01	-0.11 ± 0.01	-0.09 ± 0.01	-	-
sniffing → side by side	-0.07 ± 0.01	-0.08 ± 0.01	0.01 ± 0.01	-0.04 ± 0.01	DS>SD*(113)	DS>SD***(200)
sniffing → digging	-0.11 ± 0.01	-0.12 ± 0.01	-0.11 ± 0.00	-0.08 ± 0.01	-	DS<SD**(24)
sniffing → sniffing	0.19 ± 0.02	0.19 ± 0.02	0.14 ± 0.02	0.18 ± 0.02	-	-
